# Digital Epidemiology

**DOI:** 10.1371/journal.pcbi.1002616

**Published:** 2012-07-26

**Authors:** Marcel Salathé, Linus Bengtsson, Todd J. Bodnar, Devon D. Brewer, John S. Brownstein, Caroline Buckee, Ellsworth M. Campbell, Ciro Cattuto, Shashank Khandelwal, Patricia L. Mabry, Alessandro Vespignani

**Affiliations:** 1 Center for Infectious Disease Dynamics, Penn State University, University Park, Pennsylvania, United States of America; 2 Department of Biology, Penn State University, University Park, Pennsylvania, United States of America; 3 Department of Public Health Sciences, Karolinska Institutet, Stockholm, Sweden; 4 Interdisciplinary Scientific Research, Seattle, Washington, United States of America; 5 Harvard Medical School and Children's Hospital Informatics Program, Boston, Massachusetts, United States of America; 6 Center for Communicable Disease Dynamics, Department of Epidemiology, Harvard School of Public Health, Boston, Massachusetts, United States of America; 7 Institute for Scientific Interchange (ISI) Foundation, Torino, Italy; 8 Office of Behavioral and Social Sciences Research, NIH, Bethesda, Maryland, United States of America; 9 College of Computer and Information Sciences and Bouvé College of Health Sciences, Northeastern University, Boston, Massachusetts, United States of America; University of California San Diego, United States of America

## Abstract

Mobile, social, real-time: the ongoing revolution in the way people communicate has given rise to a new kind of epidemiology. Digital data sources, when harnessed appropriately, can provide local and timely information about disease and health dynamics in populations around the world. The rapid, unprecedented increase in the availability of relevant data from various digital sources creates considerable technical and computational challenges.

This is an “Editors' Outlook” article for *PLoS Computational Biology*.

Epidemiology, literally the “study of what is upon people”, is concerned with the dynamics of health and disease in human populations. Research in epidemiology aims to identify the distribution, incidence, and etiology of human diseases [1] to improve the understanding of the causes of diseases and to prevent their spread. Traditionally, epidemiology has been based on data collected by public health agencies through health personnel in hospitals, doctors' offices, and out in the field. In recent years, however, novel data sources have emerged where data are frequently collected directly from individuals through the digital traces they leave as a consequence of modern communication [2] and an increased use of electronic devices.

The communication revolution—the explosion of mobile phone and Internet usage—unfolding in the past few decades has led to two major outcomes: that all types of modern communication are now digital, and that the number of users of devices enabling digital communication is in the billions, rapidly approaching full coverage in large parts of the world [3]. As a consequence, an increasingly large fraction of what we do and say—including epidemiologically relevant behaviors such as deciding on preventive measures and treatment choices, as well as reporting disease symptoms—is stored electronically, often in accessible form and thus amenable to analysis. Extracting meaningful information from this data deluge is challenging, but holds unparalleled potential for epidemiology. The observation of the spatiotemporal movements of millions of people during disease outbreaks [4], the rapid detection of an unusual respiratory illness in a remote village anywhere on the globe [5], the near real-time estimation of influenza activity levels [6], [7], and the assessment of vaccination sentiments during pandemic preparedness efforts [8] are examples of realizations of this potential.

Web-based data mining is having a revolutionary impact on the way we monitor global health outcomes and behaviors. Some types of infectious and chronic disease data can be captured from and disseminated in near real-time through an array of online sources including chat rooms, social networks, blogs, web search records, and online news media. These online sources provide a picture of global health that is often different [9] from the picture created by traditional surveillance systems. In fact, these data streams have become invaluable data sources for a new generation of public health surveillance systems that operate across international borders, fill in gaps in public health infrastructure, and complement existing traditional surveillance systems [10], [11]. While for many of the most vulnerable countries, lab and clinical surveillance capacity are still years from being realized, health information is already being exchanged via web queries, social networking sites, and mobile devices.

These data sources, when harnessed appropriately, can provide local and timely information about disease outbreaks and related events around the world. Further, these sources have been credited with decreasing the time between an outbreak and formal recognition of an outbreak [12], thus allowing for an expedited response to the public health threat. Data from search engines can now provide early warning of respiratory illnesses in local communities while data from social networking sites can provide early warning of vaccine refusal stemming from conspiracy theories or other reasons. Online news media can provide a window into the emergence of pandemics weeks before it is brought to light by traditional surveillance. Similarly, data from social media could tell us about emerging trends in a wide range of health behaviors—e.g., the uptake of new tobacco products—at the local and national level.

Traditional surveillance methods emerged in a world that was very different from an epidemiological perspective. Prior to the introduction of vaccines, most deaths were caused by infectious diseases (see [13] for the vital statistics rates in the United States). In the past, networks on which diseases spread were much more limited geographically in their expansion, due to limited social and spatial mobility. This was also manifested by slower geographic dissemination of diseases [14], [15]. Nowadays, non-communicable diseases are by far the main cause of illness and death in high-income countries, while accounting for about half of the burden of disease in low- and middle-income countries [16]. Depression, type-II diabetes, and cardiovascular and pulmonary diseases pose a substantial public health risk and are typically associated with behavioral risk factors [17]. These risk factors—such as drug abuse, smoking, and poor diet and exercise—and the associated diseases are often found to be clustered in the population [18]. The processes by which this empirical pattern arises are currently not fully understood, but as more individual health behaviors and outcomes are shared online, digital epidemiology offers an increasingly clear picture of the dynamics of these processes. With respect to infectious diseases, newly emerging pathogens can appear unexpectedly, spread very rapidly, and be potentially devastating to millions. A consequence of this change in the epidemiologic landscape is that individual behaviors are now at the center of disease dynamics and control. Individual behaviors will play a key role in social distancing efforts as early responses to newly emerging, rapidly spreading infectious diseases. One of the key advantages of online social media data, apart from the increasingly large data volumes, is that they are highly contextual and networked [8], and increasingly hyperlocal (Figure 1). For example, the networked nature of the Twitter data in the vaccination sentiment study by Salathé and Khandelwal [8] allowed for the identification of network clusters with strong sentiment bias, and of positive dyadic assortativity of vaccination sentiments across the entire network of users. Overall, these advantages allow us to study individuals and groups in the rich contexts in which their lives unfold, and to study person-to-person spread of disease and behaviors at the level at which it actually occurs.

**Figure 1:**
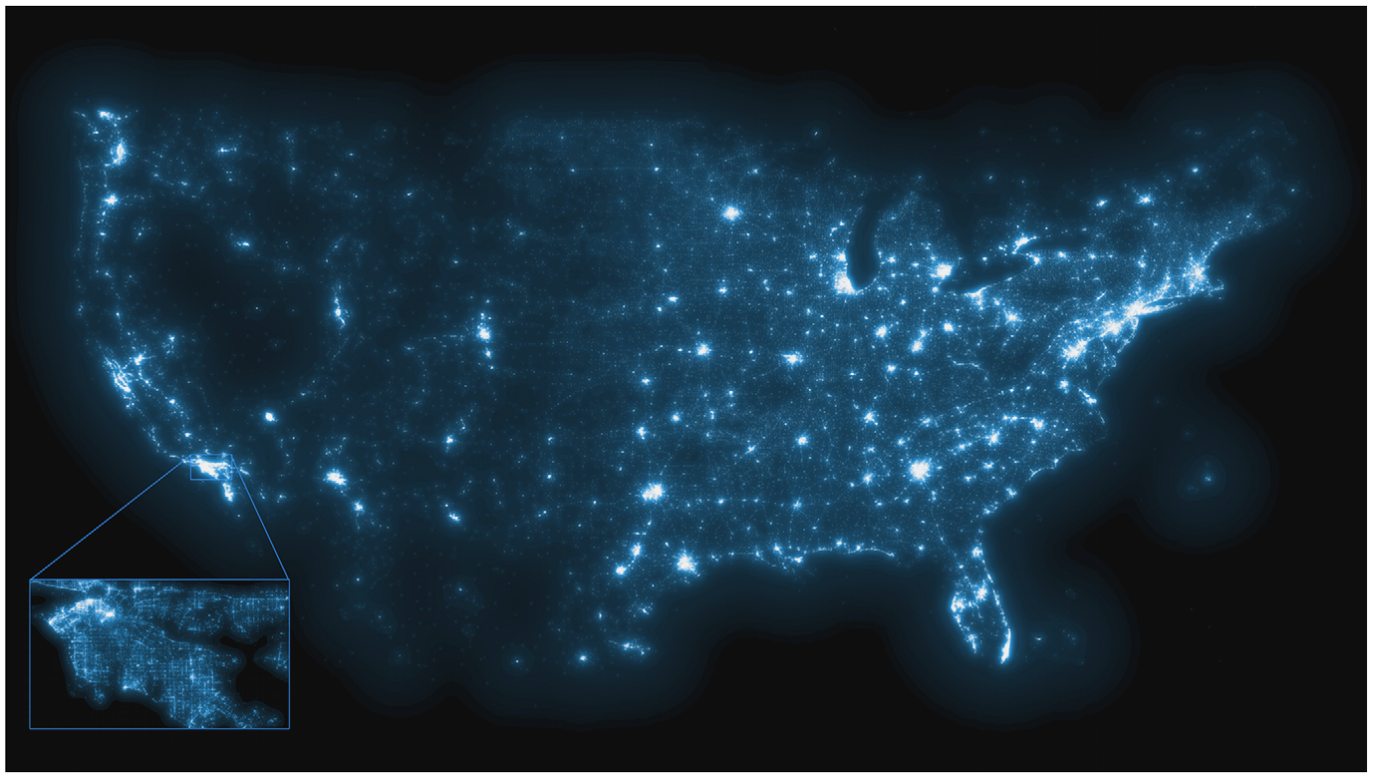
Map generated by more than 250 million public tweets (collected from Twitter.com) with high-resolution location information, broadcast between March 2011 and January 2012. Inset shows greater Los Angeles area. Brightness of color corresponds to geographic density of tweets.

Thus, there is great potential to deepen understanding of disease dynamics through the analysis of digital traces. To date, however, most epidemiologic studies involving such data have focused on presumed routes of transmission that have not yet been empirically established. For instance, we are still in need of data that indicate the relative importance of droplet, airborne, and contact transmission of influenza and other common respiratory infections in natural community settings [19]. Until researchers employ rigorous and sensitive study designs for identifying modes of transmission with confidence [20], the empirical basis of prevention advice for many infectious diseases will remain weak. Fortunately, diverse types of digital trace data may enhance exposure measurement and facilitate strong tests of specific routes of transmission. For example, in studies of small communities, such as schools or workplaces, individuals could carry or wear digital devices that sense their face-to-face proximity to others as well as record their spatial movements. Proximity and spatial mobility data, when coupled with regular surveillance of symptoms and incident infections and viral sequencing, may often distinguish modes of transmission in particular cases. Persons with genetically related infections who had no face-to-face proximity during the period when any of them were symptomatic would imply that transmission did not occur by droplets. Other combinations of proximity and spatiotemporal overlap for persons with genetically related infections would imply other modes of transmission.

The everyday movements of humans create the dynamic links that connect populations and enable geographic spread and sustained transmission of infectious diseases. Difficulties in measuring these types of human movements, traditionally estimated using travel surveys, road networks, or small-scale global positioning system (GPS) studies, have long hindered efforts to understand these dynamics. Mobile phone data in the form of call data records (containing information about the location of the mobile phone tower used during a call from a mobile phone) provide one of today's most exciting opportunities to study human mobility [21] and its influence on disease dynamics. Analogously, advances in wearable devices have radically improved our capability to track human contacts at high spatial and temporal resolution [22], affording a much more detailed characterization and understanding of social behaviors [23], complementing previous work based on large-scale surveys and self-reported information [24]. Objective measurements of social contact and mobility networks complement self-reported data and pave the way to a more accurate description of infectious disease dynamics. In particular, high quality data are needed to improve parameterization of large-scale computer simulation disease models. The introduction of these models has enabled us to broaden the traditional modeling perspective to encompass large numbers of individuals, rather than population aggregates. Mobile phone data have already been used to create realistic models of human mobility [21], predict the rate of spread of drug resistance [25], assess the prospects of malaria eradication [26], and monitor population movements during the Haiti cholera outbreak in near real-time [4]. Models based on recorded sequences of human contacts can inform the design of containment measures and of targeted immunization strategies [27] and marks an important departure from the static representation of contact networks [28]. Large-scale mobility data can be used to map the worldwide circulation of emerging infectious diseases such as the 2009 H1N1 pandemic [29], [30]. In other words, data are increasingly shaping the development of computer simulations that create *in silico* experiments hardly feasible in real systems with the goal of providing better scenario analysis for the policy making process and crisis management.

The technical challenges in all these efforts are significant. The collection, storage, and analysis of massively large data sets is made through the interface of infrastructure, software, and sophisticated algorithms. The infrastructure requirements include high bandwidth, low-latency computer networks, access to vast amounts of storage, and the availability of large clusters of machines for computation. Through state-of-the-art innovations in the cloud computing industry, unparalleled computational power and storage resources can be leased on-demand and economically. Given the real-time, large-scale demands of scientific data today, data collection and storage software need to run continuously, impervious to hardware, software, and network failure. A further challenge is the design of algorithms and data structures that are efficient and scalable for processing, mining, and analyzing dynamic and large-scale epidemiologic data. This requires the adaptation of current algorithms to run on computer clusters (cloud or dedicated), and the development of new algorithms that leverage emerging data processing techniques such as MapReduce, a programming model for processing large data sets in parallel on large distributed computer systems [31]. In addition, the extraction of knowledge (e.g., filtering, classification, anomaly detection) requires cutting-edge data mining algorithms specifically designed for the context of epidemiology. These challenges require a new breed of practitioners, combining epidemiologic expertise, analytical expertise, and advanced computational skills. They also require a curiosity to keep up with the fast pace at which novel communication tools are adopted. The adoption of social media services by hundreds of millions of people in just a few years is staggering to contemplate. At the same time, novel data streams always require careful consideration of potential biases. For example, a recent Pew Internet study [32] of Twitter users in the United States found significant differences among age groups, race/ethnicity groups, and among geographic locations.

Finally, challenges regarding data access, data sharing, and privacy need our constant attention. Some of the electronic traces that we leave as digital citizens are meant to be public, while others are not, resulting in ethical and legal challenges [33]. Furthermore, while it is easy to imagine the potential benefits of extracting information from big data, access to such data is often limited, costly, or altogether impossible for many in the research community [34]. There is also substantial variability in the sharing of data after it has been analyzed, an issue that is particularly problematic when dealing with very large data volumes because numerous—often subjective—filters need to be applied to make the original, noisy data suitable for analysis. What's more, while some data sources are legally accessible, their sharing is often not. These challenges notwithstanding, we believe that a digital epidemiology will on balance have substantial societal benefits due to the great improvements in the speed, scope, and focus of information available for public health purposes. And indeed, while these challenges still remain significant obstacles, web- and phone-based data mining is already having immediate impact on the operational activities of public health agencies worldwide.

**Authors' Biographies**

**Marcel Salathé** is an Assistant Professor of Biology at the Pennsylvania State University, adjunct faculty at the Computer Science and Engineering Department, and a Society in Science – Branco Weiss fellow. His research group at the Center for Infectious Disease Dynamics (CIDD) is interested in how health and disease dynamics are affected by the interaction of biological and social diffusion processes. He's the co-creator of crowdbreaks.com, a crowd-sourced disease surveillance system using social media data.

**Linus Bengtsson** is a medical doctor at the Division of Global Health at Karolinska Institute, Stockholm, Sweden. He is co-founder of the non-profit initiative Flowminder.org. His research focuses on uses of mobile phone operator data to follow population movements during natural disasters and infectious disease outbreaks in order to improve efficiency of relief operations.

**Todd Bodnar** is a computational biologist at Penn State University who is interested in the spread of disease and behavior with in human populations. His recent work lies at the nexus of machine learning and agent-based modeling.

**Devon D. Brewer**, PhD, is the director of Interdisciplinary Scientific Research (http://www.interscientific.net) in Seattle, United States of America. He conducts research in the health and social sciences on social networks, research methods and design, infectious disease, substance abuse, crime and violence, and other diverse topics.

**John Brownstein**, PhD, is an Associate Professor at Harvard Medical School and directs the Computational Epidemiology Group at the Children's Hospital Informatics Program in Boston. His research is dedicated to statistical and informatics approaches aimed at improving public health practice. He has been at the forefront of the development and application of public health surveillance, including HealthMap.org, an Internet-based participatory global infectious disease intelligence system.

**Caroline Buckee's** work focuses on the population dynamics of genetically diverse pathogen species including the malaria parasite and the meningococcus. She uses a range of modeling techniques to understand the relationship between the evolution of these species and the epidemiological patterns of infection and disease among human populations.

**Ellsworth Campbell** is a University Graduate Fellow and doctoral student in the Salathé Research Group at the Center for Infectious Disease Dynamics. His research employs theoretical models of human behavior and infectious disease to aid in the development of rational public policy.

**Ciro Cattuto** is a senior researcher at the ISI Foundation in Turin, Italy. His initial training is in computational statistical physics. He has recently worked on measuring, analyzing, and modeling human contact networks in hospitals and schools, as well as on computational investigations of epidemic processes over time-dependent complex networks.

**Shashank Khandelwal** is a software developer at the Center for Infectious Disease Dynamics at Penn State University. He designs and implements big data software systems to solve interesting problems at the intersection of biology and computer science. He has recently worked on creating a disease surveillance system based on open-source social media data.

**Patricia L. Mabry** is a behavioral scientist and a Senior Advisor in the Office of Behavioral and Social Sciences Research at the National Institutes of Health. She has devoted the past several years to facilitating the adoption of systems science approaches (including a variety of modeling and simulation methods) by the behavioral and social science research community. In particular, she has developed training institutes, educational videocasts, and funding and cross-disciplinary networking opportunities for those pursuing health research questions.

**Alessandro Vespignani** is the Sternberg Distinguished Professor at Northeastern University in Boston, where he leads the Laboratory for the Modeling of Biological and Socio-technical Systems. He is fellow of the American Physical Society, member of the Academy of Europe, and fellow of the Institute for Quantitative Social Sciences at Harvard University. He is also serving in the board/leadership of a variety of journals and the Institute for Scientific Interchange Foundation. He is now focusing his research activity in modeling diffusion phenomena in complex systems, including the realistic and data-driven computational modeling of infectious diseases spread.
